# Neoblast-enriched zinc finger protein FIR1 triggers local proliferation during planarian regeneration

**DOI:** 10.1007/s13238-018-0512-0

**Published:** 2018-03-20

**Authors:** Xiao-Shuai Han, Chen Wang, Fang-hao Guo, Shuang Huang, Yong-Wen Qin, Xian-Xian Zhao, Qing Jing

**Affiliations:** 1 Key Laboratory of Stem Cell Biology, Institute of Health Sciences, Shanghai Jiao Tong University School of Medicine & Shanghai Institutes for Biological Sciences, Chinese Academy of Sciences, Shanghai, 200025 China; 2 Department of Cardiology, Changhai Hospital, Shanghai, 200433 China

**Keywords:** local proliferation, adult stem cells, *Dis3l2*, wound recognition, planarians, Schmidtea mediterranea

## Abstract

Regeneration, relying mainly on resident adult stem cells, is widespread. However, the mechanism by which stem cells initiate proliferation during this process *in vivo* is unclear. Using planarian as a model, we screened 46 transcripts showing potential function in the regulation of local stem cell proliferation following 48 h regeneration. By analyzing the regeneration defects and the mitotic activity of animals under administration of RNA interference (RNAi), we identified factor for initiating regeneration 1 (*Fir1*) required for local proliferation. Our findings reveal that *Fir1*, enriched in neoblasts, promotes planarian regeneration in any tissue-missing context. Further, we demonstrate that DIS3 like 3′-5′ exoribonuclease 2 (*Dis3l2*) is required for *Fir1* phenotype. Besides, RNAi knockdown of *Fir1* causes a decrease of neoblast wound response genes following amputation. These findings suggest that *Fir1* recognizes regenerative signals and promotes *DIS3L2* proteins to trigger neoblast proliferation following amputation and provide a mechanism critical for stem cell response to injury.

**Electronic supplementary material:**

The online version of this article (10.1007/s13238-018-0512-0) contains supplementary material, which is available to authorized users.

## Introduction

Regeneration is a common phenomenon throughout the animal kingdom. For example, in mammals, hair follicle and epidermis can regenerate following injury (Fuchs and Segre, 2000; Seifert et al., 2012), and some invertebrates such as *Hydra* are capable of whole-animal regeneration from tissue pieces (Govindasamy et al., 2014; Sanchez Alvarado, 2000). There are two general regeneration groups: epimorphosis, which comprises all cases of regeneration that involve proliferation to form new tissue, and morphallaxis, in which regeneration can occur in the absence of cell proliferation (Morgan, 1901). The source of proliferative cells varies among the organisms exhibiting epimorphic regeneration. Adult stem cells (ASCs), residing in adult tissues, are undifferentiated cells and divide to replenish senescent cells and regenerate wounded tissues (Beachy et al., 2004; Clarke et al., 2000). The proliferation of ASCs is essential to initiate regeneration. It is reported that many signaling pathways are involved in the regulation of adult stem cell proliferation. For example, the transforming growth factor-β signaling is implicated in the control of muscle stem cell proliferation during adult skeletal muscle regeneration (Carlson et al., 2008), while canonical Wnt signaling promotes the proliferation of peripheral olfactory stem cells during the peripheral olfactory regeneration (Wang et al., 2011). However, these signals come from extrinsic molecules, the intrinsic regulators that govern adult stem cell proliferation *in vivo* remain largely elusive.

Planarians are a classical model for studying regeneration, as they can regenerate their whole bodies after amputation even from little pieces (Morgan, 1898; Reddien and Sanchez Alvarado, 2004). This amazing regenerative capacity relies on a population of adult stem cells named neoblasts (Reddien and Sanchez Alvarado, 2004), which are constantly dividing to replenish all cell types in intact animals (Newmark and Sanchez Alvarado, 2000; Pellettieri and Sanchez Alvarado, 2007). Neoblasts proliferate following wounding and are the source of new cells for regeneration (Best et al., 1968). Upon amputation, neoblasts display two waves of proliferating response: one commencing 6–8 h following wounding, whereby proliferation increases throughout the body, followed by another occurred 40 h later, in which proliferation is restricted to the wounds (Wenemoser and Reddien, 2010). The first wave is triggered following all injury types, while the second wave is specific to ‘missing-tissue’ response (Wenemoser and Reddien, 2010; Wurtzel et al., 2015). Many genes were mainly expressed in neoblasts and could regulate neoblast proliferation during regeneration. For example, *Smed-hp1-1* triggers neoblast proliferation by inducing the expression of *Mcm5* (Zeng et al., 2013). However, *Smed-hp1-1* is required for all neoblasts proliferation, not specifically for proliferation near the wounds. The intrinsic regulatory mechanisms of neoblasts that promote local proliferation responding to wound are poorly understood. Following amputation, a class of wound-induced genes was activated directly within neoblasts (e.g., *runt-1* and *cdc25-1*) (Wenemoser et al., 2012). Moreover, a recent study has revealed that all kinds of injury activate a common wound-response transcriptional progress, and neoblasts express most wound-induced genes (Wurtzel et al., 2015). These findings suggest that neoblasts play critical roles in response to early injury. Nevertheless, the neoblast intrinsic genes controlling neoblast wound response are needed to be identified.

In the present study, we identified that *Fir1* was required for local proliferation response for regeneration. *Fir1* is mainly expressed in neoblasts and promotes regeneration following amputation. Further, we found that *Dis3l2* is required for *Fir1* phenotype. Moreover, the expression of neoblast wound response genes is reduced in *Fir1*(RNAi) animals following amputation. These results suggest that *Fir1* senses regenerative signals and promotes *DIS3L2* proteins to trigger neoblast proliferation following amputation and provide a mechanism critical for neoblast response to injury.

## Results

### Identification of *Fir1* required for local proliferation by screening

**Figure 1:**
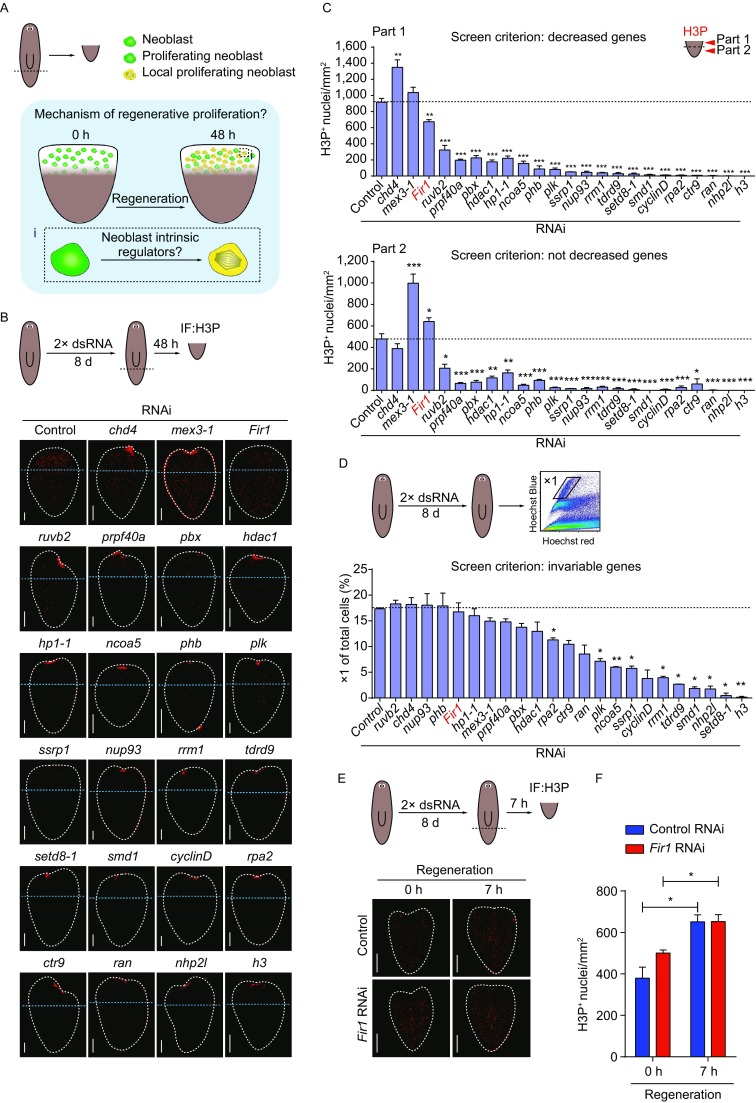
***Fir1*** **is required for local neoblast mitosis**. (A) The scientific question needed to be addressed. The mitosis adjacent to the wounds is specifically induced by tissue-missing injury (Wenemoser and Reddien, 2010), while neoblast intrinsic regulators involved in this process are still unknown. (B) Representative confocal projections through tail pieces fixed 48 h post-amputation following RNAi administration, stained with H3P antibody. The wound surfaces of tail pieces are up. Dotted lines (white): tail piece boundary. Dotted lines (blue) separate the tail pieces into two parts for quantification in (C). Unless otherwise noted, animals were fed 2× dsRNA and amputated as indicated in the cartoon (dotted red lines). Scale bars, 100 μm. (C) Mitotic density in part 1 and part 2 as separated in (B). Only one gene, *Fir1*, satisfies our screen criterions. In *Fir1*(RNAi) tail pieces mitotic density in part 1 reduced significantly, while in part 2 it was not deceased. Error bars represent SEM; * equals *P* < 0.05; *** equals *P* < 0.0001; significance determined with Student’s *t* test. (D) *Fir1* RNAi did not affect neoblast number (percentage of X1 cells) as assayed by flow cytometry. Error bars represent SEM; * equals *P* < 0.05; ** equals *P* < 0.001; significance determined with Student’s *t* test. (E) Representative confocal projections through tail pieces fixed 0 h and 7 h post-amputation following RNAi knockdown of *Fir1*, stained with H3P antibody. Dotted lines: tail piece boundary. (F) Quantification of H3P staining in (E). These results indicate that *Fir1*(RNAi) do not affect the general response of neoblasts to amputation. Error bars represent SEM; * equals *P* < 0.05; significance determined with Student’s *t* test

We aimed to identify neoblast intrinsic regulators required for local proliferation and explore the mechanisms underlying their function (Fig. 1A). In mammals, the proliferation of adult stem cells is essential for regeneration. Thus, considering the findings reported in the extant literature, we hypothesized that if there exist neoblast intrinsic regulators required for local neoblast proliferation, these genes could strongly promote planarian regeneration. First, we searched published papers for reports on neoblast regulators and phenotypic transcription factors screened by RNA interference (RNAi) and aimed to identify them in our lab. Considering the strength of regenerative phenotype upon RNAi and the expression pattern, we finally chose 46 genes, which were reported to strongly promote planarian regeneration and be enriched in neoblasts, as candidates for screening (Almuedo-Castillo et al., 2014; Blassberg et al., 2013; Bonuccelli et al., 2010; Böser et al., 2013; Chen et al., 2013; Gonzalez-Estevez et al., 2012; Guo et al., 2006; Hollenbach et al., 2011; Labbe et al., 2012; Li et al., 2011; Onal et al., 2012; Oviedo and Levin, 2007; Palakodeti et al., 2008; Rossi et al., 2007; Rouhana et al., 2010; Salvetti et al., 2005; Sanchez Alvarado, 2000; Scimone et al., 2010; Solana et al., 2012; Wagner et al., 2012; Wenemoser et al., 2012; Zayas et al., 2005; Zeng et al., 2013; Zhu et al., 2015; Zhu and Pearson, 2013) (Table S1 and Fig. S1A). In the next phase of our study, we examined regeneration phenotypes of these genes after two rounds of dsRNA feeding and found that trunk pieces showed severe regeneration defects following each of 23 genes RNAi (Fig. S1B). We randomly chose 6 genes to validate their RNAi efficiency, and we found that the gene expression reduced to about 30% following each of 6 genes RNAi, suggesting that our RNAi is efficient and has no off-target (Fig. S1C). In addition, we stained the tail pieces 48 h post-amputation using an antibody recognizing phosphorylated histone H3 at serine 10 (H3P) upon RNAi knockdown of each of the 46 candidates. Our observations revealed that mitotic activity decreased significantly at this time point after inhibition of most genes, indicating that many neoblast regulators could control neoblast proliferation generally (Fig. 1B). To confirm this observation, we quantified mitoses in each of two defined regions: adjacent to the wound sites (part 1) and the remaining tail piece (part 2). Indeed, we found that the mitotic density decreased dramatically following inhibition of each of 23 genes in both regions, such as *ruvb2*, *prpf40a*, *pbx*, *hdac1*, etc. (Fig. 1C). We also found that the mitotic events after RNAi knockdown of two genes (*chd4* and *mex3-1*) changed differently (Fig. 1C). *chd4*(RNAi) caused mitoses to increase dramatically at part 1 with no change at part 2, while *mex3-1*(RNAi) caused mitoses to increase dramatically at part 2 with no change at part 1, consistent with their main function in neoblast differentiation (Fig. 1C). Interestingly, we identified a new gene, *Fir1*, knockdown of which caused mitoses to diminish significantly at the wound sites, without affecting remaining tail pieces (Fig. 1C). To explore the delicate change of mitoses in *Fir1*(RNAi) tail pieces, we quantified the mitotic density from the wound sites and found that *Fir1*(RNAi) tail pieces displayed reduced mitotic neoblasts in the region of 150 μm away from the wounds (Figs. 1B and S1D). Furthermore, we examined neoblasts in the vicinity of wound sites in *Fir1*(RNAi) tail pieces 48 h post-amputation, and as was expected, the HP1-1^+^ and the SMEDWI-1^+^ neoblast population were reduced in *Fir1*(RNAi) animals (Fig. S1E). These results demonstrate that *Fir1* was required for local proliferation near the wounds.

If these 23 genes mentioned above mainly play roles in maintaining the local proliferation 48 h post-amputation, they should not affect the neoblast population in homeostasis (Gavino et al., 2013; Wenemoser and Reddien, 2010). Therefore, we examined ‘X1’ population (referred to as neoblasts in FACS) after inhibiting each of the representative 23 genes respectively, and found that the percentage of ‘X1’ population was comparative to the control after inhibiting 13 genes, indicating that at this time point these genes might have no effects on neoblast maintenance in homeostasis (Fig. 1D). Strikingly, *Fir1* was included in these 13 genes, indicating that *Fir1*(RNAi) does not affect neoblast maintenance before amputation. Furthermore, we investigated neoblast population and mitotic activity in intact *Fir1*(RNAi) animals at different time points, and we found that the neoblast population and the mitotic density appeared indistinguishable compared to control after *Fir1* RNAi (Fig. S1F and S1G). We also found that *Fir1*(RNAi) animals displayed homeostasis defects following long-term RNAi (Fig. S1H). The number of neoblast early (*prog-1*^+^) and late (*agat-1*^+^) progenies decreased following *Fir1* RNAi (Fig. S1I). These results suggest that *Fir1* is required for neoblast differentiation in homeostasis, which was not mainly discussed in this paper.

To exclude the possibility that *Fir1*(RNAi) affects neoblast generic response 6–8 h after injury, we examined the mitotic activity 0 h and 7 h post-amputation in *Fir1*(RNAi) animals (Fig. 1E). After quantification of the mitotic density, *Fir1*(RNAi) animals displayed neoblast generic response (Fig. 1F). Taken together, these results suggest that *Fir1* specifically controls local proliferation in early regeneration.

### *Fir1* is required for missing-tissue regeneration

**Figure 2:**
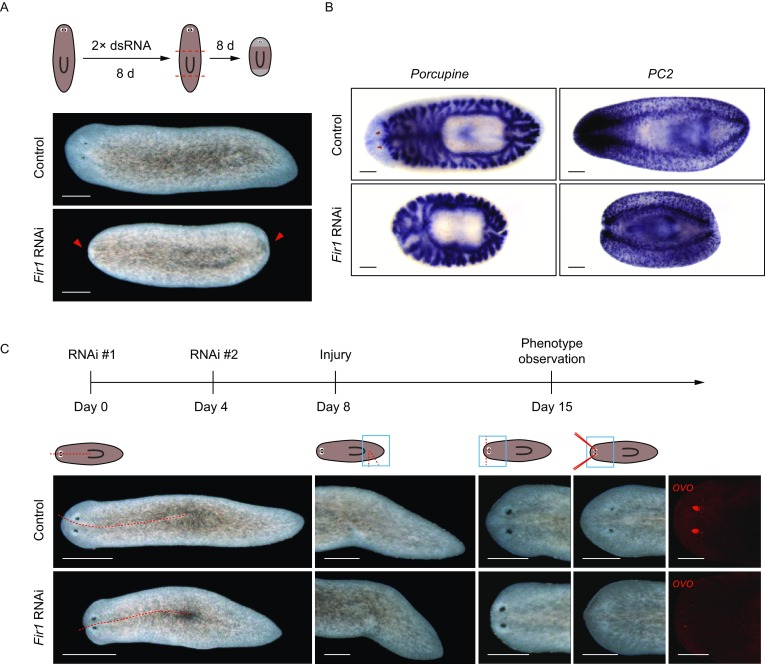
***Fir1*** **is required for missing-tissue regeneration**. (A) *Fir1*(RNAi) animals did not form blastemas after amputation (arrowheads, *n* > 100). Scale bars, 500 μm. (B) Colorimetric WISH of *Fir1*(RNAi) middle pieces following 8 days regeneration for markers of *porcupine* (intestine) and *PC2* (neurons). Scale bars, 200 μm. (C) The regeneration phenotypes after different injury types of *Fir1*(RNAi) animals. The injury included: a large incision, excision of a wedge of lateral tissue, removal of head tips and removal of eyes. *ovo* is an eye progenitor marker. For each surgical types, *n* = 6 animals. Scale bars, 500 μm

In our screening, *Fir1*(RNAi) animals could not regenerate blastemas after amputation (Figs. 2A, arrowheads, S1B and S2A). The regeneration defects were confirmed by staining *Fir1*(RNAi) trunk pieces with gastrointestinal system marker (*Porcupine*) and nervous system marker (*PC2*) 8 days post-amputation (Fig. 2B). We designed a different double-strand RNA (dsRNA) and using this dsRNA we obtained the same regeneration defects as with the dsRNA described above, indicating that *Fir1*(RNAi) phenotype is specific (Fig. S2B).

To specify the regeneration phenotype after inhibition of *Fir1*, we inflicted some other injury types to planarians, including a large incision, excision of lateral tissue wedge, removal of head tips, and removal of eyes. We observed that *Fir1*(RNAi) animals could repair the large incision, while *Fir1*(RNAi) animals were not able to regrow in other injury conditions, indicating that *Fir1* was required for missing-tissue regeneration (Fig. 2C).

### *Fir1* mRNA is predominantly expressed in neoblasts

**Figure 3:**
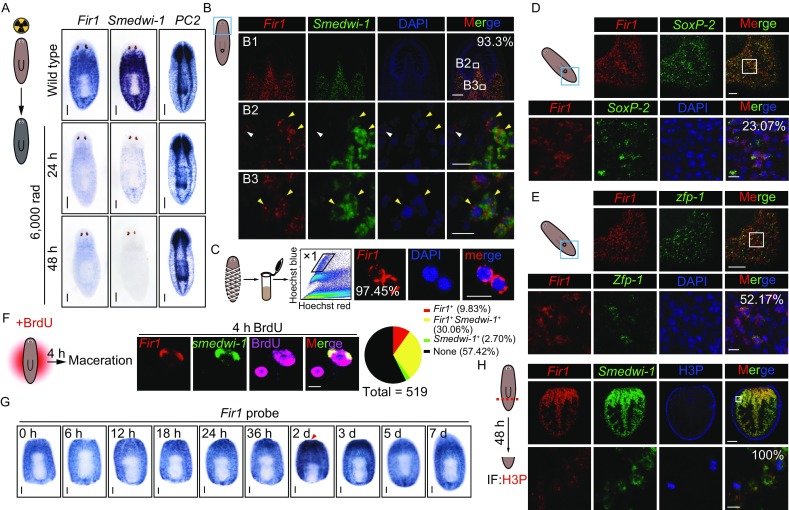
***Fir1*** **mRNA is mainly expressed in neoblasts**. (A) Colorimetric WISH of animals after 6,000 rads irradiation exposure showing that *Fir1* mRNA levels were irradiation sensitive. For each condition, *n* = 10 animals. Scale bars, 200 μm. (B) Representative confocal projections (ten 1 μm z-stacks) of planarian head, stained with *Fir1* (red), *smedwi-1* (green) and DAPI (blue). (B1) Head of the planarian. (B2) Zoom-in in the brain region. (B3) Zoom-in post brain. White arrowheads highlight *Fir1*^+^*smedwi-1*^−^ cells; yellow arrowheads point at double positive cells. The number indicates the percentage of *Fir1*^+^ cells co-expressing *smedwi-1* (*n* > 500 cells). Ventral views, anterior up. Scale bars, B1: 100 μm; B2, B3: 10 μm. (C) *Fir1* expression in neoblasts (referred to as X1 cells) sorted using FACS was detected using FISH. The number indicates the percentage of X1 cells expressing *Fir1* (*n* > 400 cells). Scale bar, 10 μm. (D) Double FISH of *Fir1* (red) and *soxP-2* (green) with DAPI (blue). Box indicates zoomed-in region. Ventral view of planarian tail. The number indicates the percentage of *Fir1*^+^ cells co-expressing *soxP-2* (*n* > 500 cells). Scale bar, upper panel: 50 μm; lower panel: 10 μm. (E) Double FISH of *Fir1* (red) and *zfp-1* (green) with DAPI (blue). Box indicates zoomed-in region. Ventral view of planarian tail. The number indicates the percentage of *Fir1*^+^ cells co-expressing *zfp-1* (*n* > 500 cells). Scale bar, upper panel: 50 μm; lower panel: 10 μm. (F) Representative confocal plane from macerated planarians subjected to a 4 h BrdU pulse and labeled by *Fir1* and *smedwi-1* FISH probes and BrdU IF. Pie chart quantifies the percentage of *BrdU*^+^ cells labeled with *Fir1* and *smedwi-1* FISH probes (*n* = 519). Scale bar, 5μm. (G) Colorimetric WISH of regenerating middle pieces for *Fir1*. Ventral views, for each time point, *n* = 10 animals. Scale bars, 200 μm. (H) Representative confocal projections of tail pieces 48 h following amputation, labelled by *Fir1* (red), *smedwi-1* (green) and H3P antibody (blue). Box indicates zoomed-in region. The number indicates the percentage of *H3P*^+^ cells co-expressing *Fir1* (*n* > 100 cells). Scale bars, upper panel: 100 μm; lower panel: 10 μm

To detect *Fir1* expression pattern, we produced *Fir1* probe and *Fir1* sense probe corresponding to the same region. The *Fir1* probe could detect *Fir1* mRNA robustly, while *Fir1* sense probe detected nothing by colorimetric whole-mount *in situ* hybridization (WISH), suggesting that our *Fir1* probe is specific to *Fir1* mRNA (Fig. S3A). By applying this probe, we found that *Fir1*, like many reported neoblast regulators, was mainly expressed in the planarian parenchyma (Fig. S3A). A lethal dose of gamma-irradiation (6,000 rad) depletes neoblasts of planarians, eliminating the ability to regenerate and to replace aged cells during homeostasis (Bardeen and Baetjer, 1904; Dubois, 1949; Scimone et al., 2010). Interestingly, 48 h after lethal irradiation, almost all *Fir1* mRNA was undetectable as *smedwi-1* mRNA, indicating that *Fir1* is expressed in neoblasts (Fig. 3A). Furthermore, we found that 93.3% of *Fir1*^+^ cells co-expressed *smedwi-1* mRNA, while little *Fir1* mRNA was expressed in *prog-1*^+^ or *agat-1*^+^ cells by double fluorescent *in situ* hybridization (dFISH) (Figs. 3B, S3B and S3C). *Fir1* mRNA was also expressed in the nervous system by detecting *Fir1* and *PC-2* FISH probes (Fig. S3D). In addition, we performed cell FISH on ‘X1’ cells sorted using FACS (Hayashi et al., 2006) and found that 97.45% of ‘X1’ cells expressed *Fir1* mRNA which was consistent with *Fir1* and *smedwi-1* co-localization data (Fig. 3C).

Recently, it is reported that planarian neoblasts comprise two major and functionally distinct cellular compartments, denoted as zeta and sigma (van Wolfswinkel et al., 2014). By using dFISH, we detected *Fir1* mRNA with zeta class marker *zfp-1* and sigma class marker *soxP-2* and found that 23.07% of *Fir1*^+^ cells expressed *soxP-2* mRNA and 52.17% of *Fir1*^+^ cells expressed *zfp-1* mRNA, suggesting that *Fir1* may play a role in both cell types during regeneration (Fig. 3D and 3E). Furthermore, by analyzing macerated animals subjected to 4 h BrdU pulse and labeled with *Fir1* and *smedwi-1* FISH probes and BrdU IF, we found that 56% of BrdU^+^ cells were *Fir1* mRNA positive, in which most cells were also *smedwi-1* positive, indicating that dividing cells expressed *Fir1* mRNA in homeostasis (Fig. 3F).

In regeneration, qRT-PCR results showed that like *smedwi-1*, *Fir1* mRNA expression level increased significantly and reached the maximum at Day 3 post-amputation (Fig. S3E). To understand the change in the *Fir1* mRNA expression pattern during regeneration, we performed colorimetric WISH on 10 regeneration time points and found that like *smedwi-2*, *Fir1* mRNA expression dramatically increased 48 h post-amputation especially near the wounds, suggesting that *Fir1* may play important roles in this region (Figs. 3G and S3F). Further, we examined *Fir1* mRNA, *smedwi-1* mRNA and mitosis marker H3P in tail pieces 48 h post-amputation (Fig. 3H). We observed that all H3P^+^ cells expressed *Fir1* mRNA as well as *smedwi-1* mRNA, suggesting that *Fir1* may regulate local proliferation during regeneration.

### Analysis of *Fir1* downstream genes by screening

**Figure 4:**
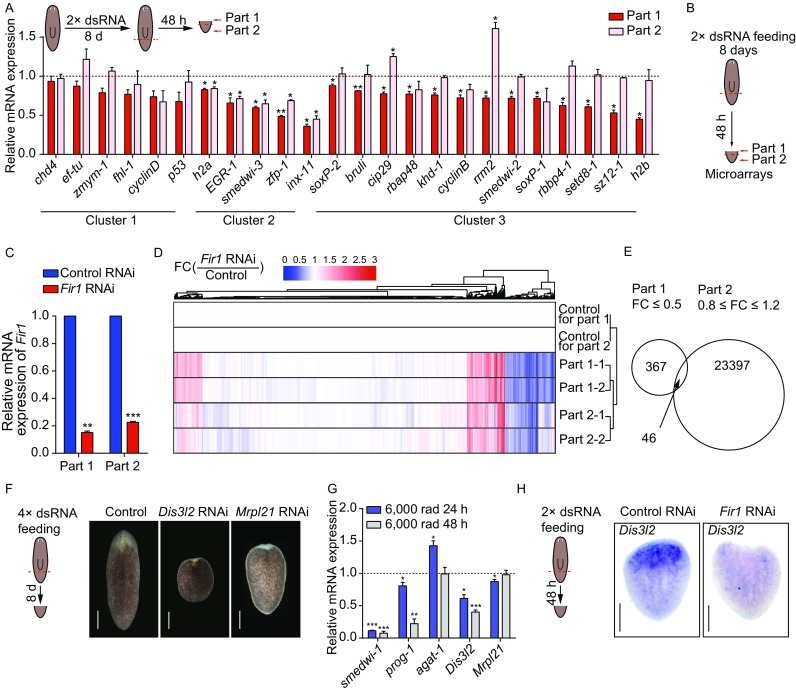
**Analysis of** ***Fir1*** **downstream genes**. (A) qRT-PCR showing the relative mRNA expression of several known neoblast regulators following *Fir1*(RNAi). Shown are averages of three independent experiments; error bars = SEM; ** equals *P* < 0.001; *** equals *P* < 0.0001; significance determined with Student’s *t* test. (B) Schematic of strategy for expression profiling. (C) qRT-PCR showing that relative expression level of *Fir1* decreased dramatically in part 1 and part 2 regions of *Fir1*(RNAi) tail pieces. Shown are averages of three independent experiments; error bars = SEM; ** equals *P* < 0.001; *** equals *P* < 0.0001; significance determined with Student’s *t* test. (D) Heat map of microarrays. Fold change (*Fir1* RNAi/control); (red) up, (blue) down; *P* < 0.05. (E) Venn diagram of genes down-regulated in the part 1 region and invariable in the part 2 region in *Fir1*(RNAi) tail piece. (F) RNAi knockdown of two candidates (*Dis3l2* and *Mrpl21*) respectively, tail pieces hardly regenerate blastemas (*n* = 6). To increase the RNAi efficiency, we chose 4× dsRNA feedings for the two genes. The animals were amputated behind the pharynx at the day after the fourth dsRNA feeding, and the tail pieces were imaged 8 days post-amputation. Scale bars, 200 μm. (G) Relative expression levels of *smedwi-1*, *prog-1*, *agat-1*, *Dis3l2* and *Mrpl21* mRNA in animals after 6,000 rads irradiation exposure. Error bars represent SEM; Student’s *t* test: * equals *P* < 0.05, ** equals *P* < 0.001, *** equals *P* < 0.0001. (H) Colorimetric WISH of *Fir1*(RNAi) tail pieces 48 h post-amputation for *Dis3l2*. For each condition, *n* = 10 animals. Scale bars, 200 μm

Considering all the results discussed above, we hypothesized that *Fir1* promotes regeneration by controlling regenerative proliferation. It is reported that many neoblast regulators could regulate planarian regeneration, we attempted to establish whether some known genes were related to the *Fir1* function (Labbe et al., 2012; Onal et al., 2012; Wagner et al., 2012). We examined the fold change of some known neoblast regulators using qRT-PCR assay in part 1 and part 2 regions following *Fir1*(RNAi) and utilized these results to classify these genes into three clusters (Fig. 4A). Genes that were not affected by *Fir1*(RNAi) were assigned to Cluster one which contained *chd4*, *ef-tu*, *zmym-1*, *fhl-1*, *cyclinD* and *p53*, and these genes were not referred to as *Fir1* downstream genes. Genes that downregulated significantly in both part 1 and part 2 belonged to Cluster two, which contained *h2a*, *egr-1*, *smedwi-3*, *zfp-1*, and *inx-11*. Although these genes were *Fir1* downstream genes, they might not participate in regulating regenerative proliferation. Genes that downregulated significantly only in part 1 constituted Cluster three, which included *soxP-2*, *bruli*, *cip29*, *rbap48*, *khd-1*, *cyclinB*, *rrm2*, *smedwi-2*, *soxP-1*, *rbbp4-1*, *setd8-1*, *sz12-1*, and *h2b*. Genes in this cluster were supposed to be downstream genes of *Fir1* controlling local proliferation. However, these genes did not pheno-copy *Fir1* upon RNAi, suggesting that *Fir1* might downregulate other new genes to drive local proliferation (Figs. 1B–D and S1B, data not shown).

In other species, the homologous of *FIR1* protein is POGZ (pogo transposable element-derived protein with zinc finger domain), which is poorly understood in stem cell research (Fig. S4A). *FIR1* protein was predicted to contain 6 ZnF_C_2_H_2_ domains from SMART, suggesting that *FIR1* may function as a transcription factor (Fig. S4B). We sought to define the molecular mechanism of *FIR1* by expression-profiling experiments. We designed custom oligonucleotide microarrays representing 61,657 predicted *S. mediterranea* transcripts and isoforms from various sources (Kao et al., 2013; Labbe et al., 2012; Onal et al., 2012; Rouhana et al., 2012; Wenemoser et al., 2012). Based on our observations that mitoses decreased dramatically at the wound sites (part 1) while increasing significantly in the remaining *Fir1*(RNAi) tail pieces (part 2) 48 h post-amputation, we isolated ‘part 1’ and ‘part 2’ tissues for microarrays (Fig. 4B). Prior to microarray analysis, we first examined the *Fir1* knockdown efficiency using qRT-PCR assay and found that *Fir1* mRNA expression decreased dramatically in both part 1 and part 2 (Fig. 4C). To display an overview of the microarray results, we clustered the transcripts according to fold change using R language, which revealed that the expression level of most transcripts did not change in either part 1 or part 2 (Fig. 4D). In addition, we checked the fold change of several known neoblast regulators and found that 25/34 of these genes showed the similar fold change as our qRT-PCR results (Figs. S4C and 4A, data not shown). According to the criterion for assignment to Cluster three above, we identified 46 genes the expression of which decreased more than 50% in part 1 and had no change in part 2 in our microarrays (Fig. 4E, Table S3). Further, considering the possible function of gene orthologs, we finally chose 26 genes for RNAi analysis, and to find more genes promoting regeneration, we increased the dsRNA feeding times to 4. Finally, we found that only *Dis3l2*(RNAi) and *Mrpl21*(RNAi) animals exhibited regeneration defects, suggesting that these two genes might function as *Fir1* downstream genes (Fig. 4F).

### *Fir1* functions through regulation of *Dis3l2*

To establish that either *Dis3l2* or *Mrpl21* functions as a *Fir1* downstream gene, we detected the mRNA expression of *Dis3l2* and *Mrpl21* by qRT-PCR in animals irradiated for 24 h and 48 h. The expression of *Mrpl21* was not reduced in animals irradiated for 48 h, indicating that *Mrpl21* was not expressed in neoblasts. In the previous study, *Fir1* was predominantly expressed in neoblasts and could control local neoblast proliferation. So, we considered that *Mrpl21* was not a direct downstream gene of *Fir1*. Interestingly, we found that *Dis3l2* mRNA expression reduced dramatically, with 50% reduction noted in samples irradiated for 48 h, indicating that *Dis3l2* was expressed in neoblasts (Fig. 4G). Planarian DIS3L2 was similar to Drosophila DIS3L2 in protein sequences and, like DIS3L2 in other organisms, it may be involved in proliferation regulation (Fig. S4D). Further, we examined *Dis3l2* mRNA expression in tail pieces regenerated for 48 h following *Fir1*(RNAi). We found that the *Dis3l2* mRNA expression decreased at the wound sites, suggesting that *Fir1* may promote *Dis3l2* expression during regeneration (Fig. 4H).

**Figure 5:**
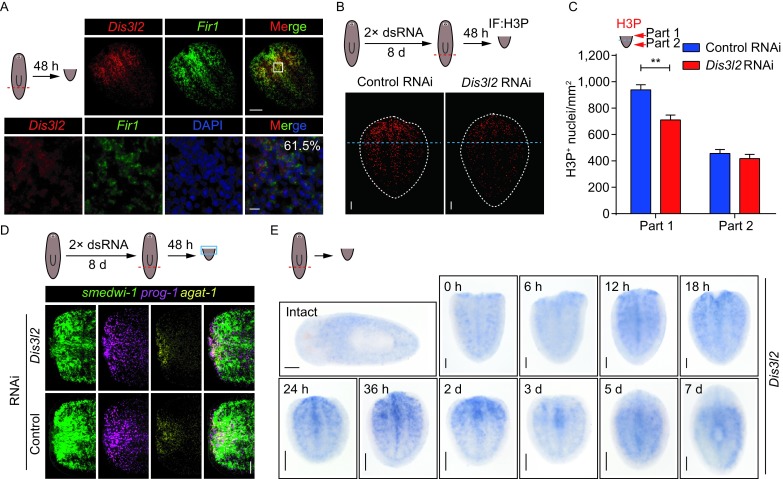
***Fir1*** **functions through regulation of** ***Dis3l2***. (A) Representative confocal projections through wildtype tail pieces fixed 48 h post-amputation, labelled with *Dis3l2* (red), *Fir1* (green) and DAPI (blue). Box indicates zoomed-in region. The number indicates the percentage of *Dis3l2*^+^ cells co-expressing *Fir1* (*n* = 561 cells). Ventral view; scale bar, upper panel: 50 μm; lower panel: 10 μm. (B) Representative confocal projections through tail pieces fixed 48 h post-amputation following RNAi administration, stained with H3P antibody. Dotted lines (white): tail piece boundary. Scale bars, 100 μm. Dotted lines (blue) separate the tail pieces into two parts for quantification in (C). (C) Mitotic density in part 1 and part 2 as separated in (B). Error bars represent SEM; ** equals *P* < 0.001; significance determined by Student’s *t* test. (D) The neoblast population adjacent of the wound sites reduced in *Dis3l2*(RNAi) tail pieces as assayed with neoblast lineage markers (*smedwi-1* for neoblast, *prog-1* for neoblast early progeny and *agat-1* for neoblast late progeny). Scale bars, 100 μm. (E) Colorimetric WISH of intact animals and regenerating middle pieces for *Dis3l2*. For each time point, *n* = 6 animals. Scale bars, 200 μm

Since the colocalization of *Fir1* and *Dis3l2* is a prerequisite of the hypothesis that *Fir1* controls local proliferation through *Dis3l2*, *Dis3l2*^+^*Fir1*^+^ cells should exist at the wound sites. To ascertain whether *Fir1* mRNA and *Dis3l2* mRNA were colocalized at the wound sites, we conducted dFISH with *Fir1* and *Dis3l2* probes in tail pieces that have been regenerating for 48 h and noted that 61.5% of *Dis3l2*^+^ cells also expressed *Fir1* mRNA at the wound sites (Fig. 5A). We also examined the expression pattern in intact animals, and we observed that *Dis3l2* was expressed in the planarian parenchyma, suggesting that *Dis3l2* may participate in neoblast regulation (Fig. S5A). Importantly, we detected the proliferation in *Dis3l2*(RNAi) tail pieces 48 h post-amputation and found that *Dis3l2*(RNAi) tail pieces displayed reduced mitoses at the wound sites only (Fig. 5B and 5C). To explore the delicate change of mitoses in *Dis3l2*(RNAi) tail pieces, we quantified the mitotic density from the wound sites and found that *Dis3l2*(RNAi) tail pieces displayed reduced mitotic neoblasts in the region 250 μm away from the wounds, which is wider than that of in *Fir1*(RNAi) tail pieces, suggesting that *Dis3l2* may promote local proliferation more extensively (Figs. S1D and S5B). Further, we examined neoblast lineage markers (neoblast marker *smedwi-1*, neoblast early progeny marker *prog-1* and neoblast late progeny marker *agat-1*) in *Dis3l2*(RNAi) tail pieces 48 h post-amputation. We observed that the number of *smedwi-1*^+^ cells reduced, while the number of *prog-1*^+^ and *agat-1*^+^ cells at the wound sites did not change compared to control animals (Fig. 5D). The expression of *Dis3l2* is reduced near the wound in *Fir1*(RNAi) tail pieces 48 h post-amputation, meanwhile, the accumulation of *smedwi-1*^*+*^ cells in the same region also disappeared (Fig. S5C). These results suggest that *Dis3l2 as* a downstream gene of *Fir1* is required for local proliferation.

To further validate this assertion, we examined the expression pattern of *Dis3l2* mRNA by colorimetric WISH in intact animals and regenerating tails. We observed that the expression of *Dis3l2* mRNA at the wound sites increased from 18 h to 2 days post-amputation with quite a low expression in intact worms, indicating that *Dis3l2* was induced by tissue-missing injury (Fig. 5E). Taken together, these data suggest that *Fir1* drive local proliferation, at least partially, by promoting *Dis3l2* expression.

### *Fir1* is required for neoblast wound recognition during regeneration initiation

**Figure 6:**
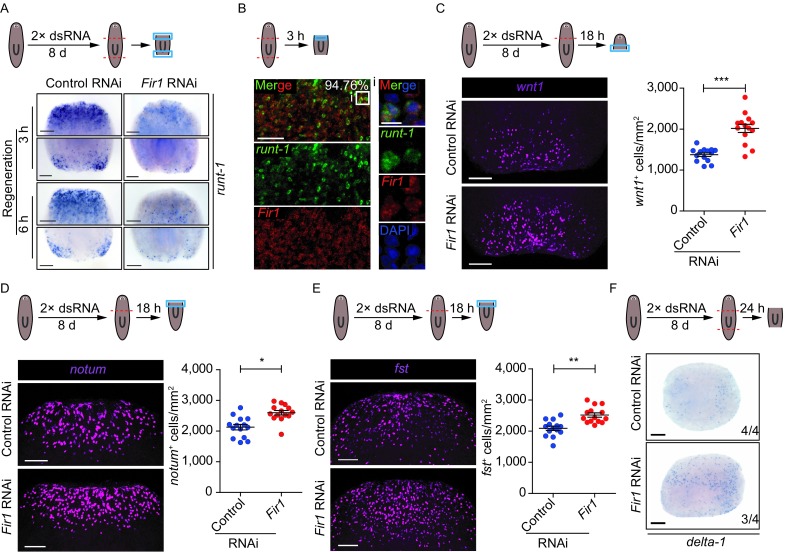
***Fir1*** **is required for neoblast wound recognition during regeneration initiation**. (A) *Fir1*(RNAi) trunk pieces displayed reduced *runt-1*^+^ cells at the wound sites 3 h and 6 h after amputation (*n* = 10/10). (B) Representative confocal projections through trunk pieces fixed 3 h post-amputation, labelled with *runt-1* (green), *Fir1* (red) and DAPI (blue). The images shown indicate the region near the anterior wound site. Box indicates zoomed-in region. The number indicates the percentage of *runt-1*^+^ cells co-expressing *Fir1* (*n* = 210 cells). Ventral view; scale bar, upper panel: 50 μm; lower panel: 10 μm. (C) *Fir1*(RNAi) head pieces displayed increased *wnt1*^+^ cells at the wound sites 18 h after amputation (*P* < 0.0001, two-tailed *t* test). (D) *Fir1*(RNAi) animals displayed increased *notum*^+^ cells at the wound sites 18 h following head amputation (*P* < 0.05, two-tailed *t* test). (E) *Fir1*(RNAi) animals displayed increased *fst*^+^ cells at the wound sites 18 h following head amputation (*P* < 0.001, two-tailed *t* test). (F) *Fir1*(RNAi) animals increased wound-induced expression of *delta-1* 24 h after amputation (*n* = 3/4). Scale bars, (A, C, D and E) 100 μm, (B and F) 200 μm

*Fir1* mRNA is mainly expressed in neoblasts that are distributed throughout the animal body, whereas, *Fir1* could control the local proliferation in regeneration. To address this question, we focused on the roles of *Fir1* in wound response to tissue absence. It is reported that many genes could be induced post-amputation, and are classified into four categories (W1, W2, W3 and W4) (Wenemoser et al., 2012). After the injury, W1 genes are activated within 30 min to 1 h and reach maximum expression by 3 h, and W2 and W3 genes are induced within 3 h to 12 h with expression peaking at 6 h. W2 genes are mostly expressed subepidermally at the wound sites, and W3 gene expression, in contrast, occurs in the epidermis far from wounds. W1, W2 and W3 genes are induced in differentiated tissues, while W4 genes are up-regulated in neoblasts. We first examined the mRNA expression of *runt-1* belonging to W4, which were highly induced in neoblasts 3–6 h post-amputation and observed that *Fir1*(RNAi) trunk pieces displayed significantly reduced *runt-1*^+^ cells, suggesting that *Fir1*(RNAi) reduced the wound recognition capacity of neoblasts (Fig. 6A). We also examined another W4 gene, *cdc25-1*, in *Fir1*(RNAi) head pieces 6 h post-amputation, and found that no evidence of *cdc25-1* expression at the wound sites, which confirmed the assertion made above (Fig. S6A, arrowheads). Furthermore, we examined *Fir1* and *runt-1* expression pattern in animals 3 h post-amputation and found that 94.76% *runt-1*^+^ cells expressed *Fir1* (Fig. 6B). These results suggest that *Fir1* is required for neoblast wound response.

Moreover, we explored genes belonging to W1, W2 and W3 in *Fir1*(RNAi) regenerating pieces. The mRNA expression of W1 genes like *jun-1* and *egrl-1* decreased 1–3 h post-amputation in *Fir1*(RNAi) animals (Fig. S6B and S6C). Notably, amputated *Fir1*(RNAi) animals displayed a higher level of expression of some W2 genes (*wnt1*, *notum* and *fst*) than did controls 18 h after amputation, suggesting that the regenerative signals were blocked (Fig. 6C–E). The expression of some W3 genes, such as *delta-1*, also increased 24 h post-amputation in *Fir1*(RNAi) animals (Fig. 6F). To further explore whether the regeneration was initiated after *Fir1*(RNAi), we detected the expression pattern of *wnt1* and *notum* 48 h post-amputation in *Fir1*(RNAi) animals. We observed that *Fir1*(RNAi) animals did not establish anterior-posterior polarity, suggesting that regenerative procedure could not be activated following *Fir1*(RNAi) (Fig. S6D).

**Figure 7:**
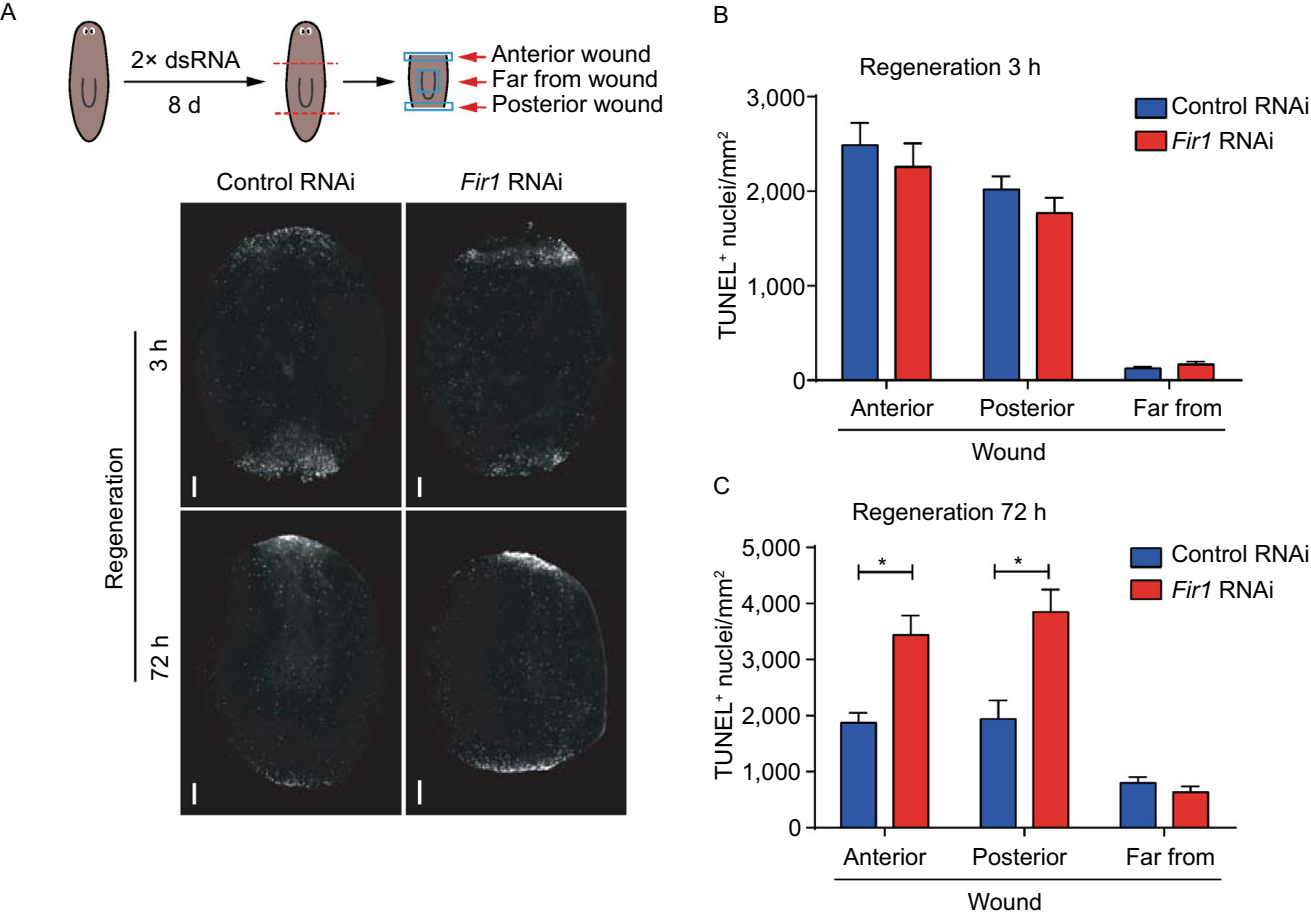
***Fir1*(RNAi) animals display abnormal apoptotic pattern during regeneration**. (A) The apoptosis in *Fir1*(RNAi) trunk pieces 3 h and 72 h post-amputation (*n* = 8/8). Scale bars, 200 μm. (B and C) Quantification of *TUNEL*^+^ cells at anterior, posterior and far from wound in *Fir1*(RNAi) trunk pieces 3 h and 72 h post-amputation respectively. Error bars represent SEM; Student’s *t* test: * equals *P* < 0.05

On the other hand, wound activates apoptosis, which could induce proliferation and provide a wounding signal in tissue repair (Fuchs and Steller, 2015). In planarians, apoptosis increases following injury and this increase involves a generic injury phase and a missing-tissue-specific phase. A local apoptosis burst initially occurs at the wound sites 3 h following any injury, and is followed by a body-wide apoptosis burst that commences 72 h after the injury, but only in cases involving missing tissue (Gavino et al., 2013; Pellettieri et al., 2010). To check if the apoptosis in response to amputation was normal during planarian regeneration, we measured apoptosis 3 h and 72 h post-amputation in *Fir1*(RNAi) animals by TUNEL. The *Fir1*(RNAi) animals displayed the same apoptosis pattern as the controls 3 h post-amputation, indicating that apoptosis for wound response was not affected by *Fir1*(RNAi) (Fig. 7A and 7B). Strikingly, 72 h post-amputation, a greater number of cells undergoing apoptosis at the wound sites was noted in *Fir1*(RNAi) animals compared to the controls, suggesting that regeneration did not initiate upon *Fir1*(RNAi) (Fig. 7A and 7C). When considered jointly, these results suggest that neoblasts at the wound sites could not recognize the regenerative signals and initiate regeneration in *Fir1*(RNAi) animals after amputation.

**Figure 8:**
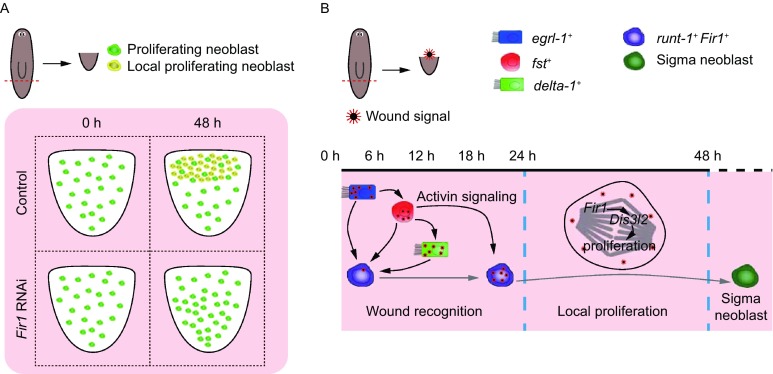
**Model of** ***Fir1*** **mechanisms in regeneration**. (A) Model of *Fir1* function in regeneration. *Fir1*, a neoblast-enriched regulator, controls regenerative proliferation. (B) A proposed genetic model for *Fir1* in regeneration. Once a missing-tissue injury occurs, *Fir1*^+^ cells at the wound sites acquire wound and regenerative signal, sense regeneration on demand. Then *Fir1* promotes the expression of *Dis3l2*, which triggers local proliferation, and generate sufficient neoblast for sequential regeneration

## Discussion

### The proliferation of adult stem cell in regeneration

In mammals, the proliferation of adult stem cells is essential for tissue/organ regeneration. For example, cardiomyocyte proliferation is critical for heart regeneration in neonatal mice (Porrello et al., 2011), and intestinal stem cell proliferation, regulated by Hippo pathway, plays an important role during Drosophila adult midgut regeneration (Shaw et al., 2010). Inhibition of cell proliferation blocks the regeneration of oral structures in the anthozoan cnidarian (Passamaneck and Martindale, 2012). In planarians, regeneration is mediated by neoblasts, the only dividing cells, which are responsible for tissue regeneration (Wagner et al., 2011). Planarian can regenerate any part of the body rapidly, which makes them an ideal model for studying stem cell function *in vivo* (Gentile et al., 2011; Sanchez Alvarado, 2003). After amputation, two phases of proliferation occur: the first is initiated after all kinds of injury, while the second is triggered only when regeneration is required (Wenemoser and Reddien, 2010). The second phase of proliferation is restricted to the wounds (also known as local proliferation), which is equivalent to the proliferation of adult stem cells for tissue/organ regeneration in mammals.

### Neoblast-enriched *Fir1* triggers local neoblast proliferation

To find neoblast intrinsic regulators required for local neoblast proliferation, we analyzed the regeneration phenotypes, the mitotic activity and the percent of neoblasts after inhibiting each of the 46 genes that were selected according to the severity of RNAi phenotypes and neoblast-enriched features reported in pertinent literature, as well as found in our proprietary database (Figs. S1B and 1B–D, Table S1). Despite the small size of our screening, we found that a novel gene *Fir1* meets our requirements exactly. First, *Fir1*(RNAi) trunk pieces cannot regenerate blastemas (Figs. S1B and 2A). Second, *Fir1*(RNAi) tail pieces display reduced mitotic activity near the wounds 48 h post-amputation (Fig. 1B and 1C). Third, the percent of neoblasts was indistinguishable from that found in the control animals (Fig. 1D). Moreover, almost all *Fir1*^+^ cells express *smedwi-1* (Fig. 3B). In our screening, we chose regenerating tail pieces, in which nearly all *Fir1*^+^ cells express *smedwi-1*, as the main model for screening genes required for local proliferation (Fig. 3H, data not shown). Therefore, these results suggest that *Fir1* may function as a neoblast intrinsic regulator controlling local proliferation. To date, many genes are reported to be required for neoblast proliferation during regeneration, such as *smedwi-2* (Reddien et al., 2005b), *Smed-hp1-1* (Zeng et al., 2013), *Smed-argonaute-2* (Li et al., 2011) and *Smed-p53* (Pearson and Sanchez Alvarado, 2010). However, most of these genes regulate proliferation of all the neoblast throughout animal body, and it is presently unknown if there exist neoblast intrinsic regulators specifically required for local proliferation responsible for a missing-tissue injury. Here, for the first time, we identified neoblast-enriched gene *Fir1* required for local proliferation, which provided *in vivo* mechanisms for adult stem cell proliferation during regeneration.

### *Dis3l2* is a potential target of *Fir1*

*FIR1* protein is predicted to contain 6 ZnF_C_2_H_2_ domains, which suggests that *Fir1* may function as a transcription factor. To find genes downstream of *Fir1* in regulating local proliferation, we mainly analyzed the downstream transcripts adjacent to the wounds after RNAi knockdown of *Fir1* by microarray. Indeed, the best way is isolating the neoblasts near the wounds to perform expression-profiling experiments. But we could not get enough neoblasts for microarray, and that is why we chose tissues near the wounds for microarray analysis instead. Although the expression of some reported neoblast regulators decreased dramatically, these genes did not affect the local proliferation required for regeneration as we expected, indicating that *Fir1* is one of the few genes involved in this process. Importantly, we identified a novel gene *Dis3l2* as a potential target of *Fir1*. First, *Dis3l2* is downregulated after RNAi knockdown of *Fir1* and promotes planarian regeneration (Fig. 4F and 4H). Second, *Dis3l2* is expressed in neoblasts and colocalizes with *Fir1* in the vicinity of wound 48 h post-amputation (Figs. 4G and 5A). Intriguingly, colorimetric WISH reveals that rare *Dis3l2* mRNA is detected in intact animals, while *Dis3l2* mRNA is induced adjacent to the wounds from 18 h post-amputation with obvious accumulation following 48 h of regeneration (Fig. 5E). Moreover, *Dis3l2* mRNA disappears in this region as regeneration progresses, which suggests that *Dis3l2* mRNA dynamics are consistent with its function during regeneration (Fig. 5E). Most importantly, *Dis3l2* is required for local proliferation, suggesting that *Dis3l2* may be a functional downstream gene of *Fir1* (Fig. 5B and 5C). *Dis3l2*, mutations in which cause the Perlman syndrome, is a member of a highly conserved family of exoribonucleases that degrade RNA in a 3′-5′ direction (Astuti et al., 2012). *Dis3l2* plays a critical role in RNA metabolism and is essential for the regulation of cell growth and division. For example, *Dis3l2* functions in the Lin28-mediated repression and degradation of let-7 microRNAs (miRNAs) in mouse embryonic stem cells (mESCs) (Chang et al., 2013; Ustianenko et al., 2013). Knockdown of *Dis3l2* enhances the growth of human cancer cell lines (Astuti et al., 2012). However, in planarian, inhibition of *Dis3l2* suppresses the local proliferation required for regeneration, suggesting that *Dis3l2* may degrade the mRNA transcribed by genes inhibiting neoblast proliferation adjacent to the wounds during regeneration.

### Neoblast response to injury requires *Fir1* function

In planarians, three major classes of wound-induced genes that are expressed in differentiated tissues (W1, W2 and W3 genes) and a class of genes induced in neoblasts (W4 genes) have been identified, which constitute a molecular wound response program to elicit regeneration (Wenemoser et al., 2012). We examined the expression pattern of representative genes in four categories following *Fir1* RNAi and found that the response of these genes to amputation displayed abnormally. The induction of W4 genes (*runt-1* and *cdc25-1*) and W1 genes (*jun-1* and *egrl-1*) was reduced in *Fir1*(RNAi) animals, while the induction of W2/3 genes like *wnt1*, *notum*, *fst*, and *delta-1* increased significantly (Figs. 6A, 6C–F and S6A–C), suggesting that *Fir1* is required for wound response during regeneration. It is reported that *fst*, a wound-induced gene expressed in differentiated tissues, is required for local mitosis by inhibiting Activin signaling (Gavino et al., 2013). The increase in the number of *fst*^+^ cells near wounds can be explained as the interruption of regenerative signals before neoblasts sensing them. Moreover, a recent study has revealed that neoblasts express most wound-induced genes, suggesting that neoblasts may play an important role in wound response (Wurtzel et al., 2015). Here, we for the first time identified *Fir1* as a potential neoblast intrinsic regulator controlling neoblast response to missing-tissue injury.

## Materials and methods

### Planarian culture and irradiation

In this study, sexual *Schmidtea mediterranea* CIW4 strain was used in all experiments. These planarians were maintained as described elsewhere (Newmark and Sanchez Alvarado, 2000; Sanchez Alvarado et al., 2002; Wang et al., 2016). Briefly, planarians were cultured in 1× Montjuic salts at 21°C in the dark, fed homogenized beef liver paste two times per week, and amputated for expansion. Planarians were starved for 1–2 weeks before experiments. 4–6 mm-long animals were used for RNAi and 1–2 mm-long animals were used for *in situ* hybridization. For irradiation, planarians were exposed to 6,000 rads on a GammaCell 3000 irradiator (Table S4). The animals were kindly provided by P. Newmark (University of Illinois at Urbana-Champaign/Howard Hughes Medical Institute, Urbana, IL), P. Reddien (Massachusetts Institute of Technology/Howard Hughes Medical Institute, Cambridge, MA), and N. Oviedo (University of California, Merced, Merced, CA).

### Gene cloning and RNAi

All planarian transcripts used in this study were cloned into the pMD18-T vector (Takara) from complementary DNA (cDNA) and verified by Sanger sequencing. RNA interference (RNAi) was mainly performed as described elsewhere (Rouhana et al., 2013). The template for producing dsRNA was generated by polymerase chain reaction (PCR) using primers with T7 promoters flanking on the 5′-ends. The sense and antisense RNA molecules were transcribed by T7 RNA polymerase (Promega) and annealed. The quality of dsRNA was assessed by non-denaturing agarose gel electrophoresis. 4 µg dsRNA with 20 μL beef liver was sufficient for inducing 15 animals RNAi. dsRNA for GFP was used as negative control. Generally, animals were fed dsRNA food every 3 days, and the animals were amputated 8 days after initial RNAi. We made a cartoon depicting experiment design for each figure, which described the number of RNAi treatments, time of amputations after initial RNAi, amputation position (as indicated with dotted red lines) and time of detection. For RNAi of *Dis3l2* and *Mrpl21* in Fig. 4F, animals got 4× dsRNA feedings. Unless otherwise noted, animals were fed 2× dsRNA food (Table S2).

### Whole-mount immunofluorescence

Whole-mount immunofluorescence was performed as described elsewhere (Newmark and Sanchez Alvarado, 2000; Wenemoser and Reddien, 2010). Briefly, animals were sacrificed and fixed in Carnoy’s on ice for 1.5 h, following bleaching in 6% hydrogen peroxide/methanol solution, animals were blocked and incubated with rabbit anti-H3P antibody (1:100, Millipore), and the mitotic activity was developed using anti-rabbit Alexa 488 (1:600, Invitrogen).

### Immunofluorescence on paraffin section

Tail pieces following 2 days regeneration were fixed in 4% formaldehyde for 2 h at 4°C. They were subsequently embedded in paraffin and sectioned adjacent to the wounds at 5 μm thickness. After deparaffination, antigen retrieval was performed in 0.01 mol/L citrate buffer, pH 6.0, for 20 min. Then the sections were blocked with 4% BSA and incubated in HP1-1 (1:100 dilution) and SMEDWI-1 (1:200 dilution) antibody solution (Zeng et al., 2013). HP1-1 and SMEDWI-1 were developed using rhodamine tyramide (1:2,000 dilution) and fluorescein tyramide (1:1,000 dilution), respectively. Horseradish peroxidase enzyme was inactivated for 20 min between labelings by 1% Hydrogen peroxide, in PBS containing 0.1% Triton-X100 (PBSTx). The sections were counterstained with DAPI (sigma, 1 μg/mL).

### Whole-mount * in situ* hybridization

Whole-mount* in situ* hybridizations were performed as described elsewhere (Pearson et al., 2009). Hybridized RNA probes were labeled with DIG-11-UTP (Sigma), Fluorescein-12-UTP (Sigma) or DNP-11-UTP (Perkin Elmer) and purified as described (Lapan and Reddien, 2011). Tyramide was generated by conjugation of succinimidyl esters of rhodamine, FITC, and AMCA with tyramide-HCL (Sigma) (Hopman et al., 1998). For horseradish peroxidase enzyme inactivation, animals were incubated in 154 mmol/L sodium azide for 2 h (King and Newmark, 2013; van Wolfswinkel et al., 2014). Animals were counterstained with DAPI (Sigma, 3 μg/mL in PBSTx) for 1 h and mounted for imaging.

### Fluorescence-activated cell sorting

The procedures of fluorescence-activated cell sorting were mainly performed as described elsewhere (van Wolfswinkel et al., 2014). Planarians were diced with a razor blade on ice-cold dishes, and the tissue mash was collected in CMFB (400 mg/L NaH_2_PO, 800 mg/L NaCl, 1,200 mg/L KCl, 800 mg/L NaHCO_3_, 240 mg/L glucose, 1% BSA, 15 mmol/L HEPES pH7.3) supplemented with 1 mg/mL collagenase (Sigma) (Reddien et al., 2005a). After digestion for 45 min under agitation at room temperature, cell suspensions were passed through a 35 μm cell-strainer cap (BD Biosciences), and pelleted. Then the cells were stained with Hoechst 33342 (Invitrogen) and propidium iodide and filtered again. Cells were sorted on a MoFlo (Beckman-Coulter), and Hoechst blue versus red plots were used to identify the ‘X1’ fraction that is high in DNA content (Hayashi et al., 2006).

### BrdU labeling

BrdU labeling was performed as described elsewhere (Cowles et al., 2012). Briefly, animals were treated with 0.0625% N-acetyl cysteine (NAC) for 1 min, washed 3 times quickly, and incubated for 1 h in 1× Montjuıïc salts containing 25 mg/mL BrdU (Sigma) and 3% dimethyl sulfoxide in the dark. Animals were washed 3 times and inculcated for 4 h in the dark.

### Single-cell FISH and immunofluorescence on cells

Cells from macerated animals or fluorescence-activated cell sorting were adhered to coverslips. Single-cell FISH was performed as described elsewhere (Scimone et al., 2014). For BrdU immunofluorescence after FISH, the procedures were similar to that in whole mount animals, with slight modifications: all washes were limited to 5 min, and the BrdU signal was developed using rhodamine tyramide (Newmark and Sanchez Alvarado, 2000).

### qRT-PCR

qRT-PCR was performed as previously described (Li et al., 2011; Wang et al., 2016; Zeng et al., 2013). Briefly, total RNA of the regenerating pieces was isolated using TRIZOL (Invitrogen). M-MLV Reverse Transcriptase (Promega) was used to synthesize cDNA from 1 μg of total RNA. Gene-specific primers were designed using Primer3 (http://frodo.wi.mit.edu/primer3/) (Table S2). qPCRs were performed with SYBR Green quantitative PCR master mix (Toyobo Co.) on a quantitative PCR system (7900HT Fast Real-Time PCR System, Applied Biosystems). Three biological replicates were performed for each group. The relative mRNA expression was plotted with GraphPad Prism.

### Microarray analysis

We designed custom oligonucleotide microarrays representing 61,657 predicted *S*. *mediterranea* transcripts and isoforms from various sources (Kao et al., 2013; Labbe et al., 2012; Onal et al., 2012; Rouhana et al., 2012; Wenemoser et al., 2012) at the eArray website (Agilent Technologies). ‘part 1’ and ‘part 2’ RNA was harvested with Trizol (Invitrogen) from control and *Fir1*(RNAi) tail pieces 48 h post-amputation. Two biological replicates were used. RNA was amplified and labeled with Cy3-CTP using a low RNA input fluorescent linear amplification kit (Agilent Technologies). Custom oligonucleotide expression arrays (Agilent) were hybridized, scanned and analyzed as previously described (Zeng et al., 2013). To find the downstream genes of *Fir1*, genes were considered if they met a corrected *P*-value threshold of 0.05 and were downregulated in ‘part 1’ and showed invariable in ‘part 2’ in duplicate samples. Hierarchical clustering and heat map generation were performed using R.

### Phylogenetic analysis

Protein sequence for SMED-FIR1 and SMED-DIS3L2 was aligned with its homologous proteins in other organisms using ClustalW with the default setting (Thompson et al., 1994). The result of ClustalW was imported to MEGA 4.0, in which neighbor-joining tree was generated using default settings and 1,000 bootstrap replicates.

### TUNEL

Animals were fixed and stained for TUNEL using a method described elsewhere (Pellettieri et al., 2010) with modifications: animals were bleached in formamide-bleaching solution (5% non-deionized formamide, 0.5× SSC, and 1.2% H_2_O_2_) (King and Newmark, 2013) for 4 h under bright light, after TdT reaction animals were washed 2 × 30 min at 65°C in 1 mmol/L EDTA, and the TUNEL signal was developed using FITC-tyramide solution (FITC-tyramide 1:1,000 and 0.006% H_2_O_2_ in PBS containing 0.01% Tween-20) for 20 min.

### Image acquisition, processing and quantification

Live animals and whole-mount *in situ* hybridization samples were photographed using a microscope (SteREO Discovery.V20; Carl Zeiss) equipped with a Plan Apochromat 1.0× objective and a digital microscope camera (AxioCam HRc; Carl Zeiss) automated by AxioVision Rel.4.8 software (Carl Zeiss). Confocal images were captured on a Leica SP5 confocal microscope with a 20×, 40×, or 63× objective. All the quantifications were using the Measurement program of Volocity (Perkin Elmer) and normalized by the quantified animal area. For H3P quantifications, all the mitotic events were determined by counting nuclei labeled with the anti-H3P antibody. For quantification of *notum*^+^, *wnt1*^+^ or *fst*^+^ cells, all the probe signals surrounding the nucleus in the vicinity of the wounds were calculated. For cells under apoptosis quantifications, we obtained all the TUNEL signal using confocal microscope (about fifty 1 μm stacks), then we used Volocity software (PerkinElmer) to build 3D image for these stacks and quantify TUNEL^+^ nuclei in our region of interest (ROI), and finally the number of TUNEL^+^ nuclei was normalized by the area of the upper surface of ROI.

### Statistical analysis

Results are presented as means ± SEM, and statistical analyses were performed in GraphPad Prism using the Student’s *t* test for two groups. *P* < 0.05 was considered significant.

## Electronic supplementary material

Below is the link to the electronic supplementary material.

Supplementary material 1 (PDF 4415 kb)

Supplementary material 2 (XLSX 12 kb)

Supplementary material 3 (XLSX 19 kb)

Supplementary material 4 (XLSX 21 kb)

Supplementary material 5 (XLSX 13 kb)
