# Supplementary material for: Neoblast-enriched zinc finger protein FIR1 triggers local proliferation during planarian regeneration
Source: Protein Cell. 2018 Mar 20;10(1):43–59. doi: 10.1007/s13238-018-0512-0 (PMC6321819; doi:10.1007/s13238-018-0512-0)
Supplement: Supplementary file 1 — Supplementary material 1 (PDF 4415 kb) [file 13238_2018_512_MOESM1_ESM.pdf]

**Supplemental Information**

**Figure Legends**

**Fig. S1.** Identification of *Fir1* required for regeneration-specific proliferation by screening. (A) The strategy of screening in this study. To identify genes required for local neoblast proliferation, three rules were proposed. First, these genes can strongly promote regeneration, which is guaranteed by twice dsRNA feeding and severe regeneration defects. Second, these genes are required for regenerative proliferation. Third, RNAi knockdown of these genes does not affect the neoblast population. (B) The trunk pieces can hardly regenerate blastemas following RNAi knockdown of 23 genes respectively. After dsRNA inhibition of 46 candidates, we measured the area of anterior blastemas in trunk pieces and found that each trunk piece regenerates blastemas less than 0.03 $mm^2$ following perturbation of 23 genes respectively. For each RNAi condition, $n \geq 30$ animals. Scale bars, 500 µm. (C) RNAi efficiency as assayed by qRT-PCR. Error bars represent SEM; Student's *t* test: *** equals $p<0.0001$. (D) *Fir1*(RNAi) tail pieces displayed reduced mitotic numbers in the region of 150 µm away from the wounds. Mitotic neoblasts were quantified at different distances from the wound sites in control and *Fir1*(RNAi) tail pieces 48 hours following amputation. Quantification of mitotic density is plotted from 5 animals for each position. Error bars represent SEM; ** equals $p<0.001$; significance determined with Student's *t* test. (E) The expression of HP1-1 and SMEDWI-1 reduced in the vicinity of the wounds in *Fir1*(RNAi) tail pieces. The expression pattern of HP1-1 (red) and SMEDWI-1 (green) was examined on paraffin sections by double immunofluorescence. Paraffin sections were obtained from adjacent to the wounds in control and *Fir1*(RNAi) tail pieces 48 hours following amputation. For each condition, n = 3 animals. Scale bars, 50 µm. (F) *Fir1* RNAi did not affect neoblast number (percentage of X1 cells) as assayed by flow cytometry. (G) Mitotic activity was examined 6, 8, 10 and 12 days after *Fir1* RNAi. For each condition, $n \geq 5$ animals. (H) *Fir1*(RNAi) worms did not maintain homeostasis following long-term RNAi (n > 50 animals). White arrowhead indicates the lysis of planarian head. Scale bars, 500 µm. (I) Neoblast early (*prog-1*$^+$) and late (*agat-1*$^+$) progenies were investigated 6, 8, 10 and 12 days after *Fir1* RNAi. For each condition, $n \geq 3$ animals. Error bars represent SEM; * equals p<0.05; significance determined with Student's t test.

**Fig. S2.** *Fir1*(RNAi) animals display regeneration defects. (A) *Fir1*(RNAi) tail and head pieces did not form blastemas. Animals were fed two rounds of *Fir1* dsRNA and amputated at day 8. The regeneration phenotypes of tail and head pieces were observed. Scale bars, 500 µm. (B) Specificity of the *Fir1* RNAi phenotype. Animals fed with a different dsRNA corresponding to the 5'-end of *Fir1* failed to regenerate ( n = 6 ). Scale bars, 500 µm.

**Fig. S3.** *Fir1* mRNA expression pattern. (A) Colorimetric WISH showing *Fir1* probe is specific. Dorsal view, anterior is left; scale bars, 200 µm. (B) Representative confocal projections (ten 1µm z-stacks) of the middle position in planarian, stained with *Fir1* (red) and *prog-2* (green). Box indicates zoomed-in region. Dorsal views, anterior up. The number indicates the percentage of *Fir1*$^+$ cells co-expressing *prog-2* (n > 400 cells). Scale bars, left panel: 200 µm; right panel: 50um. (C) Double FISH of *Fir1* (red) and *agat-1* (green). Box indicates zoomed-in region. Dorsal views, anterior up. The number indicates the percentage of *Fir1*$^+$ cells co-expressing *agat-1* (n > 400 cells). Scale bars, left panel: 200 µm; right panel: 50 µm. (D) Double FISH of *Fir1* (green) and *PC-2* (red) with DAPI (white). (D1) whole animal. (D2) Zoom-in in the brain region. (D3) Zoom-in nerve cord of tail. White arrowheads highlight double positive cells. Ventral views, anterior up; scale bars, D1: 100 µm; D2: 50 µm; D3: 25 µm. (E) qRT-PCR showing relative expression levels of *Fir1*, *smedwi-1*, *prog-1* and *agat-1* mRNA during regeneration. Animals were

amputated anterior and posterior of the pharynx to generate three pieces. The RNA was extracted from regenerating pieces at 10 time points during regeneration. The mRNA expression levels of *Fir1* and neoblast lineage markers (*smedwi-1*, *prog-1*, and *agat-1*) were examined in these samples by qRT-PCR. (F) *smedwi-2* expression pattern during regeneration. Colorimetric WISH of regenerating middle pieces for *smedwi-2*. Ventral views, for each time point, n = 10 animals. Scale bars, 200 μm.

**Fig. S4.** Analysis of *Fir1* downstream genes. (A) Phylogenetic analysis of the *Fir1* gene. Bar, 10% amino acid substitution. H. sapiens (Hs), M. musculus (Mm), R. norvegicus (Rn), G. gallus (Gg), X. tropicalis (Xt), D. rerio (Dr), D. melanogaster (Dm), C. elegans (Ce), A. thaliana (At), S. pombe (Sp), S. cerevisiae (Sc) and S. mediterranea (Smed) are shown. (B) *Fir1* protein includes 6 ZnF_C2H2 domains from SMART prediction. *Fir1* protein sequence was acquired from Six-Frame Translation, and the conserved domains were predicted using SMART (Simple Modular Architecture Research Tool). (C) Heat map of known neoblast regulators in the microarray. The fold change of known neoblast regulators was extracted from the expression-profiling data. Heat map was generated by R. (D) Phylogenetic analysis of the *Dis3l2* gene. Bar, 10% amino acid substitution. H. sapiens (Hs), M. musculus (Mm), R. norvegicus (Rn), G. gallus (Gg), X. tropicalis (Xt), D. rerio (Dr), D. melanogaster (Dm), C. elegans (Ce), A. thaliana (At), S. pombe (Sp), S. cerevisiae (Sc) and S. mediterranea (Smed) are shown.

**Fig. S5.** *Dis3l2* serves as a functional downstream gene of *Fir1*. (A) The expression pattern of *Dis3l2* in intact animals. Colorimetric WISH of intact animals for *Dis3l2*. n = 6 animals. Scale bars, 200 μm. (B) *Dis3l2*(RNAi) tail pieces displayed reduced mitotic numbers in the region of 250 μm away from the wound sites. To explore the delicate change of mitoses in *Dis3l2*(RNAi) tail pieces, mitotic density (Figure 5B) was quantified from the wound sites. For each condition, n = 6 animals. Error bars represent SEM; Student's *t* test: * equals $p<0.05$, ** equals $p<0.001$, *** equals $p<0.0001$. (C) The accumulation of *smedwi-1*$^{+}$ cells disappeared following *Fir1*(RNAi). Representative confocal projections through tail pieces fixed 48 hours post-amputation following RNAi administration, stained with *smedwi-1* (green) and *Dis3l2* (red). Scale bars, 100 μm.

**Fig. S6.** *Fir1* is required for the expression of wound response genes. (A) *Fir1*(RNAi) head pieces had reduced wound-induced expression of *cdc25-1* at wound site 6 hours after amputation (arrows, n = 7/8). Another wound-induced neoblast response gene, *cdc25-1*, was examined by colorimetric WISH in control and *Fir1*(RNAi) animals. Scale bars, 100 μm. (B) *Fir1*(RNAi) trunk pieces displayed reduced *jun-1*$^{+}$ cells at the wound sites after amputation. Wound-induced *jun-1* was examined by colorimetric WISH in control and *Fir1*(RNAi) animals following amputation. For each condition, n = 6 animals. Scale bars, 100 μm. (C) *Fir1*(RNAi) trunk pieces displayed reduced *egrl-1*$^{+}$ cells at the wound sites after amputation. Wound-induced *egrl-1* was examined by FISH in control and *Fir1*(RNAi) animals following amputation. For each condition, n = 6 animals. Scale bars, 100 μm. (D) *Fir1*(RNAi) trunk pieces did not establish anterior-posterior polarity 48 hours following amputation (arrows, n = 6/6). Colorimetric WISH of *Fir1*(RNAi) trunk pieces 48 hours post-amputation for anterior pole marker (*notum*) and posterior pole marker (*wnt1*). Scale bars, 100 μm.

Fig. S1

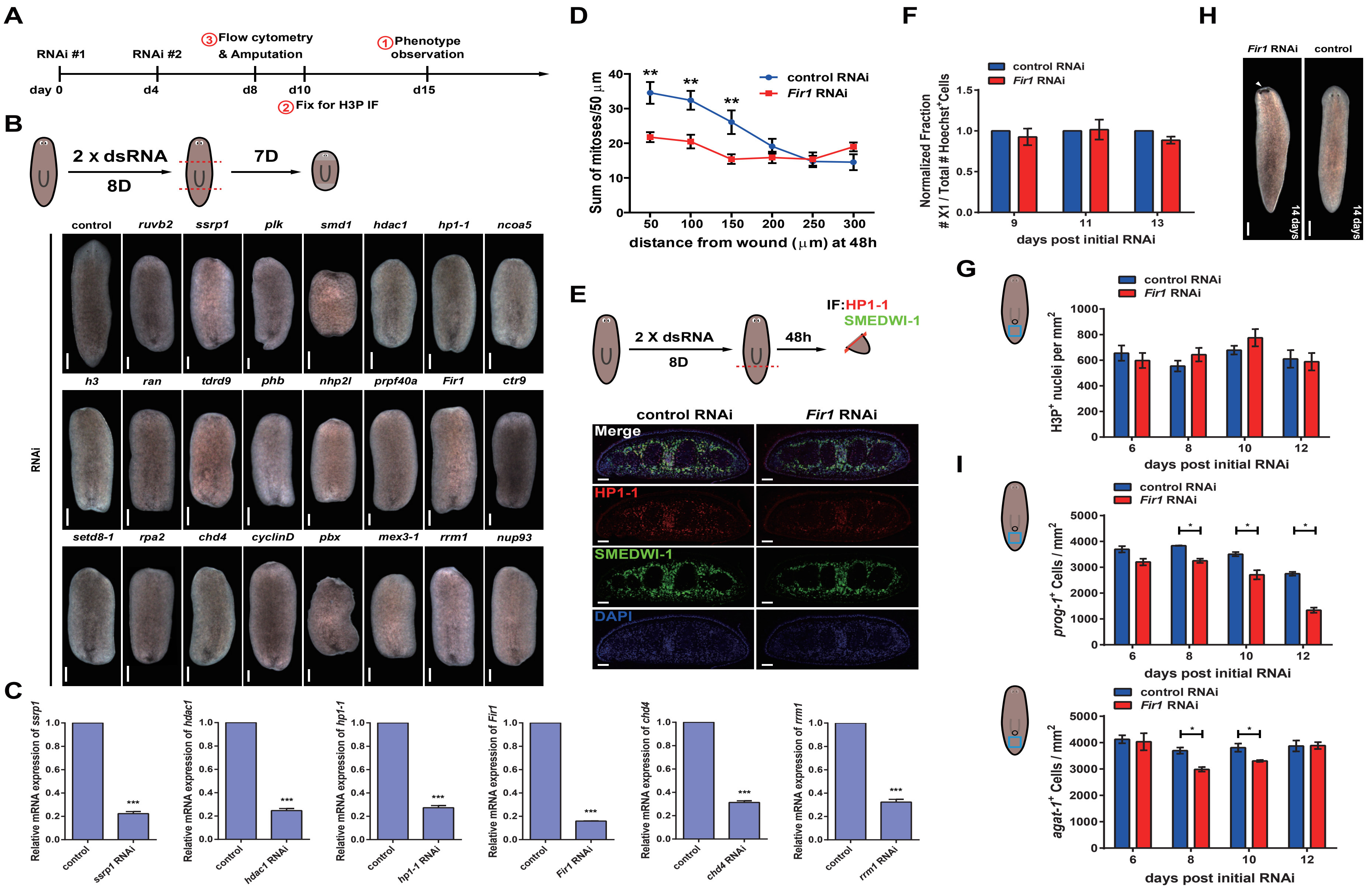
A
RNAi #1
RNAi #2
3 Flow cytometry & Amputation
1 Phenotype observation
day 0
d4
d8
d10
d15
2 Fix for H3P IF
B
2 x dsRNA
8D
7D
RNAi
control
ruvb2
ssrp1
plk
smd1
hdac1
hp1-1
ncoa5
h3
ran
tdrd9
phb
nhp2l
prpf40a
Fir1
ctr9
setd8-1
rpa2
chd4
cyclinD
pbx
mex3-1
rrm1
nup93
C
Relative mRNA expression of ssrp1
Relative mRNA expression of hdac1
Relative mRNA expression of hp1-1
Relative mRNA expression of Fir1
Relative mRNA expression of chd4
Relative mRNA expression of rrm1
control
ssrp1 RNAi
hdac1 RNAi
hp1-1 RNAi
Fir1 RNAi
chd4 RNAi
rrm1 RNAi
D
Sum of mitoses/50 µm
distance from wound (µm) at 48h
control RNAi
Fir1 RNAi
E
2 X dsRNA
8D
48h
IF:HP1-1
SMEDWI-1
control RNAi
Fir1 RNAi
Merge
HP1-1
SMEDWI-1
DAPI
F
Normalized Fraction
# X1 / Total # Hoechst⁺Cells
control RNAi
Fir1 RNAi
days post initial RNAi
H
Fir1 RNAi
control
14 days
G
H3P⁺ nuclei per mm²
days post initial RNAi
I
prog-1⁺ Cells / mm²
agat-1⁺ Cells / mm²
days post initial RNAi

Fig. S2

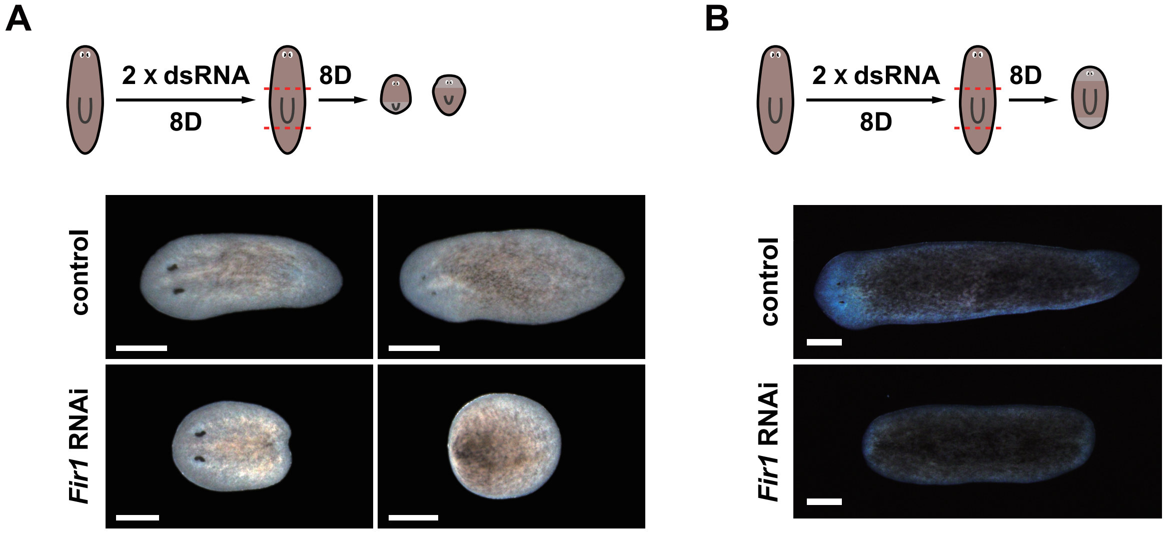
A
2 x dsRNA
8D
8D
control
Fir1 RNAi
B
2 x dsRNA
8D
8D
control
Fir1 RNAi

Fig. S3

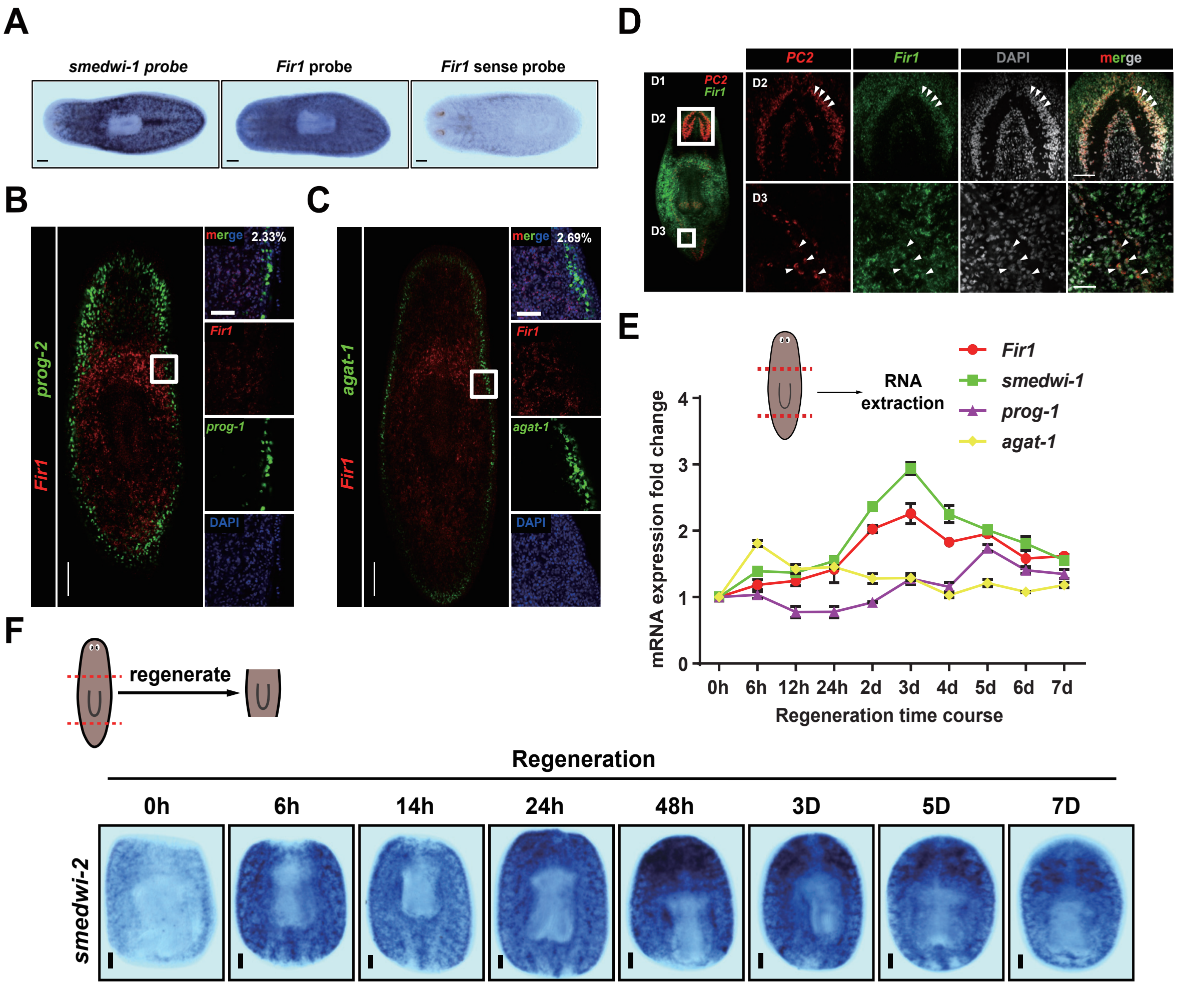
A
smedwi-1 probe
Fir1 probe
Fir1 sense probe
B
prog-2
Fir1
merge 2.33%
Fir1
prog-1
DAPI
C
agat-1
Fir1
merge 2.69%
Fir1
agat-1
DAPI
D
PC2
Fir1
DAPI
merge
D1
D2
D3
E
RNA extraction
Fir1
smedwi-1
prog-1
agat-1
mRNA expression fold change
0h 6h 12h 24h 2d 3d 4d 5d 6d 7d
Regeneration time course
F
regenerate
Regeneration
0h
6h
14h
24h
48h
3D
5D
7D
smedwi-2

Fig. S4

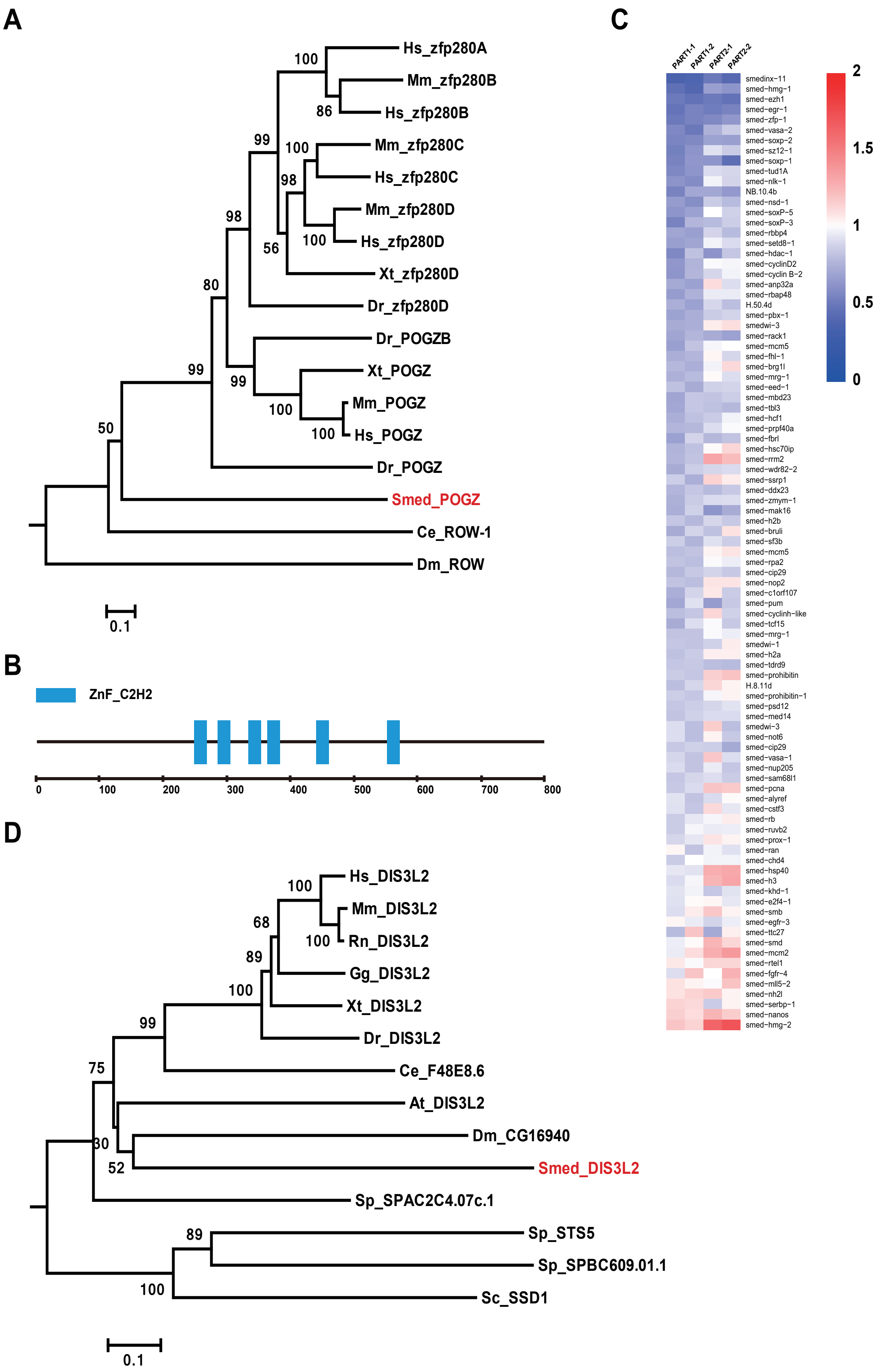

A
Hs_zfp280A
Mm_zfp280B
Hs_zfp280B
Mm_zfp280C
Hs_zfp280C
Mm_zfp280D
Hs_zfp280D
Xt_zfp280D
Dr_zfp280D
Dr_POGZB
Xt_POGZ
Mm_POGZ
Hs_POGZ
Dr_POGZ
Smed_POGZ
Ce_ROW-1
Dm_ROW
100
86
99
100
98
100
56
98
80
99
99
100
100
50
0.1
B
ZnF_C2H2
0
100
200
300
400
500
600
700
800
C
PART1-1
PART1-2
PART2-1
PART2-2
smedinx-11
smed-hmg-1
smed-ezh1
smed-egr-1
smed-zfp-1
smed-vasa-2
smed-soxp-2
smed-sz12-1
smed-soxp-1
smed-tud1A
smed-nlk-1
NB.10.4b
smed-nsd-1
smed-soxP-5
smed-soxP-3
smed-rbbp4
smed-setd8-1
smed-hdac-1
smed-cyclinD2
smed-cyclin B-2
smed-anp32a
smed-rbap48
H.50.4d
smed-pbx-1
smedwi-3
smed-rack1
smed-mcm5
smed-fhl-1
smed-brg1l
smed-mrg-1
smed-eed-1
smed-mbd23
smed-tbl3
smed-hcf1
smed-prpf40a
smed-fbrl
smed-hsc70ip
smed-rrm2
smed-wdr82-2
smed-ssrp1
smed-ddx23
smed-zmym-1
smed-mak16
smed-h2b
smed-bruli
smed-sf3b
smed-mcm5
smed-rpa2
smed-cip29
smed-nop2
smed-c1orf107
smed-pum
smed-cyclinh-like
smed-tcf15
smed-mrg-1
smedwi-1
smed-h2a
smed-tdrd9
smed-prohibitin
H.8.11d
smed-prohibitin-1
smed-psd12
smed-med14
smedwi-3
smed-not6
smed-cip29
smed-vasa-1
smed-nup205
smed-sam68l1
smed-pcna
smed-alyref
smed-cstf3
smed-rb
smed-ruvb2
smed-prox-1
smed-ran
smed-chd4
smed-hsp40
smed-h3
smed-khd-1
smed-e2f4-1
smed-smb
smed-egfr-3
smed-ttc27
smed-smd
smed-mcm2
smed-rtel1
smed-fgfr-4
smed-mll5-2
smed-nh2l
smed-serbp-1
smed-nanos
smed-hmg-2
2
1.5
1
0.5
0
D
Hs_DIS3L2
Mm_DIS3L2
Rn_DIS3L2
Gg_DIS3L2
Xt_DIS3L2
Dr_DIS3L2
Ce_F48E8.6
At_DIS3L2
Dm_CG16940
Smed_DIS3L2
Sp_SPAC2C4.07c.1
Sp_STS5
Sp_SPBC609.01.1
Sc_SSD1
100
68
100
89
100
99
75
30
52
89
100
0.1

Fig. S5

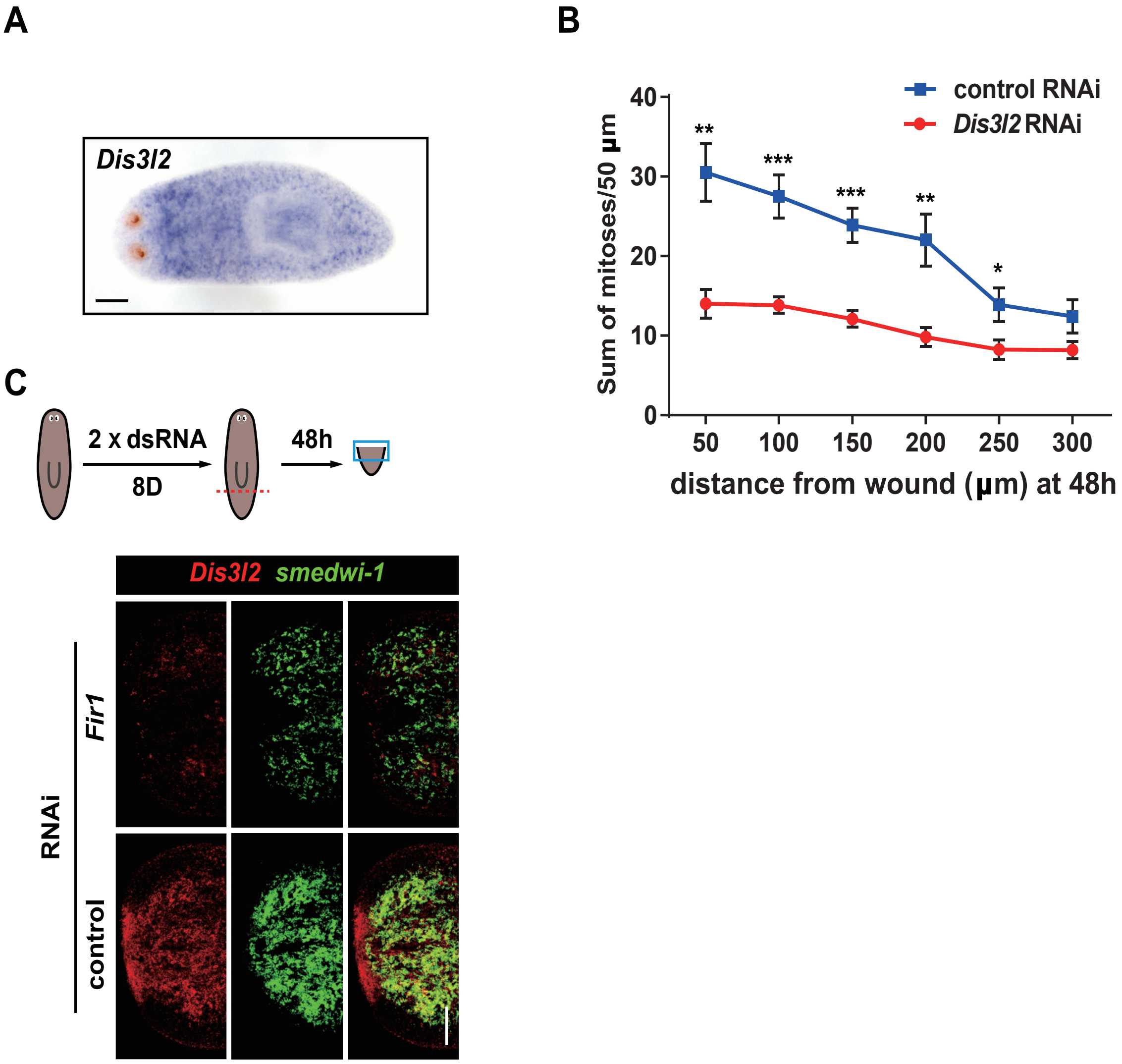
A
Dis3l2
B
control RNAi
Dis3l2 RNAi
Sum of mitoses/50 µm
40
30
20
10
0
**
***
***
**
*
50
100
150
200
250
300
distance from wound (µm) at 48h
C
2 x dsRNA
8D
48h
Dis3l2 smedwi-1
Fir1
control
RNAi

Fig. S6

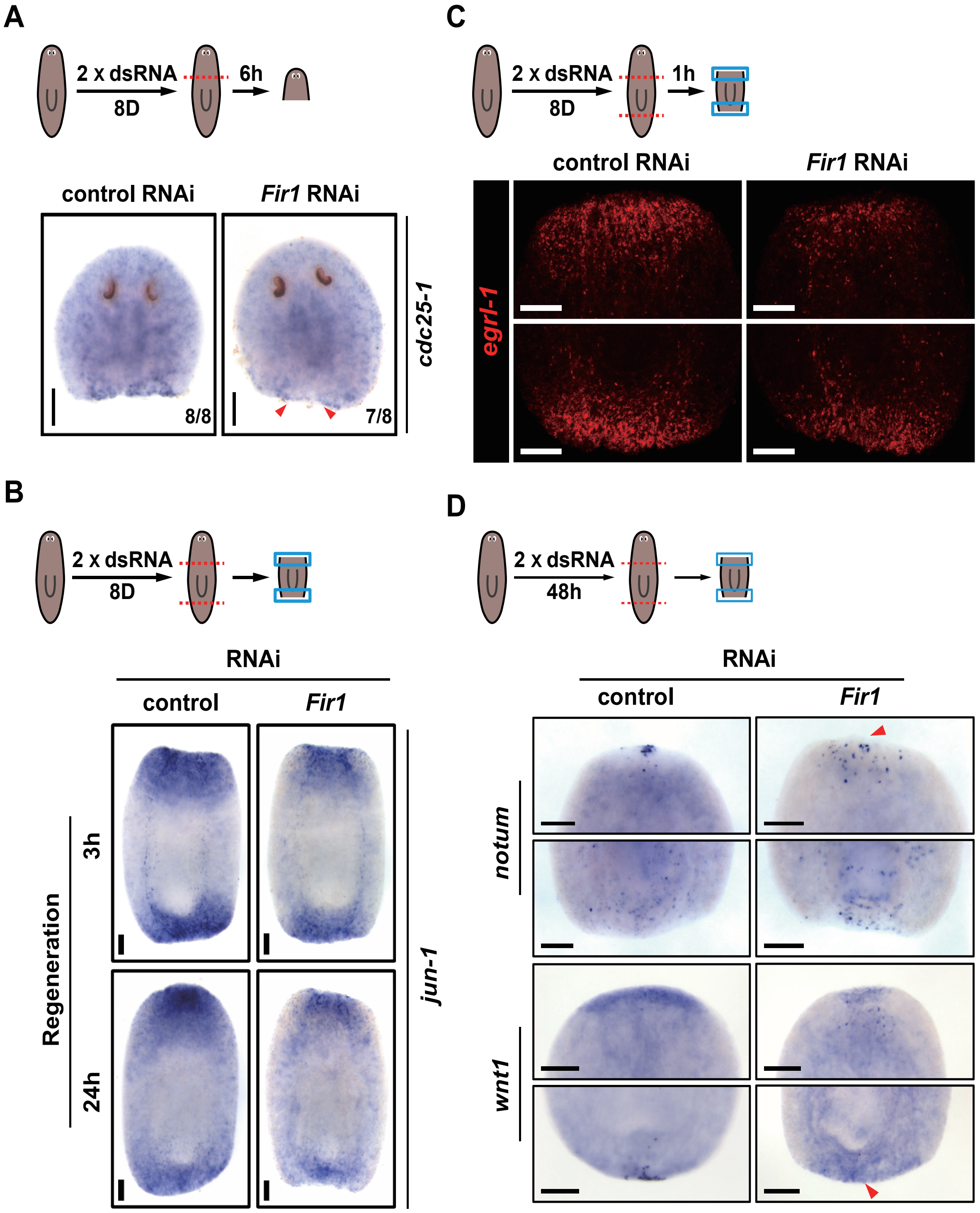
A
2 x dsRNA
8D
6h
control RNAi
Fir1 RNAi
cdc25-1
8/8
7/8
B
2 x dsRNA
8D
RNAi
control
Fir1
Regeneration
3h
24h
jun-1
C
2 x dsRNA
8D
1h
control RNAi
Fir1 RNAi
egrl-1
D
2 x dsRNA
48h
RNAi
control
Fir1
notum
wnt1
